# Comparative modelling of foetal exposure to maternal long-acting injectable versus oral daily antipsychotics

**DOI:** 10.1038/s44294-025-00077-9

**Published:** 2025-05-15

**Authors:** Philip Bediako-Kakari, Mariella Monyo, Shakir Atoyebi, Adeniyi Olagunju

**Affiliations:** 1 School of Biosciences, University of Liverpool, Liverpool, UK; 2 Centre of Excellence for Long-acting Therapeutics, University of Liverpool, Liverpool, UK; 3 Department of Biochemistry, Cell, and Systems Biology, University of Liverpool, Liverpool, UK

**Keywords:** Drug therapy, Therapeutics

## Abstract

This study employed physiologically based pharmacokinetic (PBPK) modelling to compare the extent of foetal exposure between oral and long-acting injectable (LAI) aripiprazole and olanzapine. Adult and pregnancy PBPK models were developed and validated with relevant clinical data. Relevant indices of foetal exposure during pregnancy were predicted from concentration-time data at steady-state dosing for both oral and LAI formulations. Foetal C_max_ for aripiprazole was 59–78% higher with LAI than oral, and 68–181% higher with LAI olanzapine than the oral formulation. Predicted cord:maternal ratios (range) were 0.59–0.69 for oral aripiprazole and 0.61–0.66 for LAI aripiprazole, 0.34–0.64 for oral olanzapine and 0.89–0.96 for LAI olanzapine. Also, cumulative foetal exposure over 28 days from oral formulations were generally predicted to be lower compared with their therapeutic-equivalent LAI. As *in utero* foetal exposure to maternal drugs does not necessarily translate to risk, these data should be interpreted in a broader context that includes benefit-risk assessments.

## Introduction

Around 1.5 to 3.5% of the general population will be diagnosed with a psychotic disorder, while a higher percentage will experience at least one psychotic symptom at some point in their lives (https://www.ncbi.nlm.nih.gov/books/NBK546579/). Implications of poor treatment of psychosis lead to significant repercussions not only for the affected individuals, but the whole society. Such implications could include worsening of symptoms, functional decline^1^, increased risk of suicide^2^, physical health deterioration^3^, increased hospitalisations^1^, social isolation and stigma^4^, burden on families, caregivers and healthcare services as well as legal and forensic issues^5,6^.

Oral antipsychotic drugs remain the standard treatment option for psychosis. These drugs, classified into first-generation or second-generation antipsychotics, function by altering neurotransmitter activity in the brain to reduce symptoms such as hallucinations and delusions. While oral antipsychotics are widely used, their effectiveness can be underutilized by poor adherence to medication regimens, particularly in individuals with severe mental illness who may have cognitive deficits or lack insight into their condition^7–9^. Additionally, oral medications are subject to first-pass metabolism in the liver, leading to variable bioavailability and a significant portion of the drug being metabolised before reaching systemic circulation^10,11^. This variability may necessitate higher doses to achieve therapeutic effects^12,13^, increasing the risk of side effects^14,15^.

Long-acting injectables (LAIs) were developed to address some of the limitations associated with oral antipsychotics, particularly adherence and bioavailability issues, by offering a consistent drug delivery system^16^. They ensure guaranteed administration and transparency of adherence by eliminating the need for daily dosing^17,18^. These lead to improved treatment outcomes, as LAIs also reduce the likelihood of missed doses, unintentional overdose, and abrupt relapses due to partial or overt nonadherence^19–21^. Additionally, by bypassing gastrointestinal absorption, LAIs avoid first-pass metabolism^20,21^ leading to consistency in bioavailability^22^, resulting in reduced peak-trough plasma levels^16,23^. However, LAIs also present challenges, such as slow dose titration, injection-site discomfort, and frequent visits for treatment^16^. These issues may be amplified during pregnancy, where physiological changes can alter drug disposition^24–26^.

Pregnancy presents a period of significant psychological^27,28^ and physiological change^25,26^, and for women with pre-existing mental health conditions, an increased risk of exacerbation or relapse^24^. As such, managing psychosis during pregnancy is crucial, as untreated mental illness can harm both the mother and the foetus, leading to poor prenatal care, substance abuse, and obstetric complications^29^. Pregnancy can further complicate oral medication use due to nausea, vomiting, and difficulty swallowing, particularly during the first trimester^30^. Additionally, there is a risk of inadvertent foetal exposure to antipsychotics^31^, which can be associated with low birth weight, preterm birth, and congenital malformations^32,33^. Despite these risks, continuing antipsychotics is often necessary for maternal and foetal health^33^, because discontinuing treatment may lead to deterioration in mental health status affecting the ability to function as a caregiver^34^. However, there is limited knowledge on how foetal exposure differs between oral and LAI antipsychotics and understanding these differences could help reduce the risk of congenital issues.

Estimating foetal exposure is challenging due to the ethical and practical limitations on direct sampling of foetal blood. Consequently, various indirect methods have been used to predict foetal drug levels including pharmacokinetic (PK) modelling, placental tissue sampling, cord blood sampling at delivery, and the use of animal models. Among these, physiologically based pharmacokinetic (PBPK) models have gained prominence, allowing for the simulation of *in utero* maternal-to-foetal drug transfer throughout pregnancy. These models integrate physiological changes occurring in both the mother and the foetus during gestation^35,36^, providing a dynamic approach to estimate foetal drug exposure across different stages of pregnancy^37^.

Aripiprazole (ARI) and olanzapine (OLZ) are commonly prescribed antipsychotics, targeting serotonin (5-HT) and dopamine receptors. Aripiprazole is metabolised by CYP2D6 and CYP3A4 into its active metabolite dehydro-aripiprazole, while olanzapine is metabolised mainly through CYP1A2, CYP2C8, and UGT1A4. Both drugs are available in oral once-daily tablets (aripiprazole: 2–30 mg; olanzapine: 2.5–20 mg) and LAI formulations. The LAI formulation of aripiprazole is available in two distinct forms: aripiprazole once-monthly (AOM) and aripiprazole lauroxil. AOM (Abilify Maintena®) is a monohydrate version of aripiprazole with molecular weight of 466.4 g/mol (https://www.otsuka-us.com/sites/g/files/qhldwo9046/files/media/static/Abilify-PI.pdf). Conversely, aripiprazole lauroxil (Aristada*®*) is a prodrug with a higher molecular weight (660.7 g/mol) (https://www.aristada.com/downloadables/ARISTADA-PI.pdf). AOM comes in 300 mg and 400 mg doses, equivalent to 15 mg and 20 mg daily doses, respectively^38,39^. The LAI formulation of olanzapine contains olanzapine esterified with pamoic acid which confers sustained-release properties enabling four-weekly intramuscularly (IM) administration at a dose of 300 mg or 405 mg^40^.

In this study, we used PBPK modelling to compare foetal exposure during pregnancy between oral antipsychotics and their therapeutic-equivalent LAI formulations.

## Results

### Adult models validation

The observed and simulated PK metrics, including the area under the drug concentration-time curve (AUC) and the maximum drug concentration within a specified time interval (Cmax), for both orally and intramuscularly administered aripiprazole and olanzapine were compared, as shown in Supplementary Table 1. Clearance values (CL) reported in the tables were derived using non-compartmental analysis (NCA) of the concentration-time profiles generated by the PBPK model by applying the equation CL = Dose/AUC. The PBPK model was successfully validated for both aripiprazole and olanzapine PK, with the calculated absolute average fold error (AAFE) for all parameters within the set 2-fold threshold. Likewise, predictions made by the pregnancy PBPK model were consistent with the sparse concentration measurements available in pregnant persons (Supplementary Table 2).

### Pregnancy models validation

Simulations performed to estimate foetal drug exposure successfully predicted cord plasma and maternal plasma concentrations for both aripiprazole and olanzapine within the set acceptable range, as shown in Supplementary Table 3. All calculated AAFEs were below the 2-fold threshold, thus, the PBPK model performance was considered satisfactory to simulate foetal exposure to both aripiprazole and olanzapine after maternal dose during pregnancy.

### Foetal exposure simulations

Predicted indices of foetal exposure at steady state summarised as cord-to-maternal plasma drug concentration (C:M) ratios and other predicted PK parameters of aripiprazole and olanzapine are shown in Table 1. Generally, foetal exposure was predicted to decrease with increasing gestation in pregnancy for oral olanzapine, oral aripiprazole, and LAI aripiprazole, however, foetal exposure to the two doses of LAI olanzapine was inconsistent between the second and third trimester.

**Table 1:** Predicted pharmacokinetic parameters of repeated oral versus therapeutic-equivalent LAI aripiprazole doses at steady state during pregnancy

Pharmacokinetic parameter	Second trimester (*n* = 100)	Third trimester (*n* = 100)	Second trimester (*n* = 100)	Third trimester (*n* = 100)
Aripiprazole	15 mg oral repeated doses^a^		20 mg oral repeated doses^a^	
Mean C:M ratio	0.69 (0.04)	0.60 (0.04)	0.68 (0.05)	0.59 (0.04)
mAUC_0__–24 h,ss_ (µg.h/mL)	1.54 (0.23)	1.21 (0.19)	2.00 (0.31)	1.62 (0.25)
fAUC _0__–24 h,ss_ (µg.h/mL)	1.08 (0.65)	0.75 (0.51)	1.37 (0.83)	1.00 (0.68)
mAUC_0__–28d,ss_ (µg.h/mL)	43.12 (6.44)	33.88 (5.32)	56.00 (8.68)	45.36 (7.00)
fAUC_0__–28d,ss_ (µg.h/mL)	30.24 (18.2)	22.00 (14.28)	38.36 (23.24)	28.00 (19.04)
fAUC/mAUC	0.70 (0.31)	0.62 (0.29)	0.69 (0.31)	0.62 (0.29)
mCmax_ss_ (ng/mL)	96.4 (15.7)	84.9 (16.0)	126 (21.1)	113 (21.2)
fCmax_ss,oral_ (ng/mL)	57.8 (8.80)	43.3 (7.94)	74.2 (11.7)	57.5 (10.5)
	**300** **mg LAI repeated doses**^**b**^		**400** **mg LAI repeated doses**^**b**^	
Mean C:M ratio	0.64 (0.01)	0.62 (0.01)	0.66 (0.01)	0.61 (0.01)
mAUC_0__–28d,ss_ (µg.h/mL)	78.0 (2.77)	57.0 (6.41)	104 (4.62)	75.5 (7.30)
fAUC_0__–28d,ss_ (µg.h/mL)	50.5 (21.3)	37.2 (24.9)	70.6 (35.4)	47.8 (28.4)
fAUC/mAUC	0.65 (0.24)	0.65 (0.28)	0.68 (0.29)	0.63 (0.26)
mCmax_ss_ (ng/mL)	148 (4.64)	108 (11.7)	197 (7.72)	143 (13.4)
fCmax_ss,LAI_ (ng/mL)	94.5 (16.4)	71.1 (46.1)	132 (65.7)	91.4 (53.2)
fCmax_ss,LAI_/fCmax_ss,oral_	1.64 (0.38)	1.64 (1.11)	1.78 (0.93)	1.59 (0.97)
**Olanzapine**	**10** **mg oral repeated doses**^**a**^		**15** **mg oral repeated doses**^**a**^	
Mean C:M ratio	0.64 (0.09)	0.36 (1.4)	0.62 (0.5)	0.34 (0.3)
mAUC_0__–24h,ss_ (µg.h/mL)	0.42 (0.04)	0.42 (0.04)	0.62 (0.05)	0.58 (0.07)
fAUC _0__–24h,ss_ (µg.h/mL)	0.25 (0.22)	0.15 (0.13)	0.36 (0.29)	0.20 (0.18)
mAUC_0__–28d,ss_ (µg.h/mL)	11.76 (1.12)	11.76 (1.12)	17.36 (1.40)	16.24 (1.96)
fAUC_0__–28d,ss_ (µg.h/mL)	7.00 (6.16)	4.20 (3.64)	10.08 (8.12)	5.60 (5.04)
fAUC/mAUC	0.6 (0.5)	0.36 (0.3)	0.59 (0.5)	0.35 (0.2)
mCmax_ss_ (ng/mL)	30.1 (2.9)	29.1 (3.3)	43.8 (3.7)	41.6 (4.1)
fCmax_ss,oral_ (ng/mL)	14.4 (10.3)	9.3 (6.6)	20.8 (13.9)	12.7 (8.7)
	**300** **mg LAI repeated doses**^**b**^		**405** **mg LAI repeated doses**^**b**^	
Mean C:M ratio	0.96 (0.3)	0.89 (0.3)	0.96 (0.3)	0.92 (0.3)
mAUC_0–28d,ss_ (µg.h/mL)	10.3 (1.19)	10.0 (0.74)	13.6 (0.93)	13.5 (0.89)
fAUC _0–28d,ss_ (µg.h/mL)	9.51 (2.52)	9.60 (3.07)	13.2 (4.14)	12.4 (4.28)
fAUC/mAUC	0.93 (0.2)	0.96 (0.3)	0.97 (0.3)	0.92 (0.30)
mCmax_ss_ (ng/mL)	27.1 (2.6)	27.2 (2.0)	36.5 (2.5)	36.5 (2.4)
fCmax_ss,oral_ (ng/mL)	24.7 (7.0)	26.1 (9.1)	35.0 (11.1)	33.3 (11.4)
fCmax_ss,LAI_/fCmax_ss,oral_	1.72 (0.7)	2.81 (1.4)	1.68 (0.8)	2.62 (1.3)

Data are presented as mean (SD) or mean alone.

*C:M* cord-to-maternal plasma drug concentration ratio, *mAUC*_***ss***_ maternal area under the plasma concentration-time curve under one dosing interval at steady-state, *fAUC*_***ss***_ foetal area under the plasma concentration-time curve under one dosing interval at steady-state, *mCmax*_***ss***_ maternal maximum concentration at steady-state, *fCmax*_***ss***_ foetal maximum concentration at steady state, *fAUC/mAUC* foetal to maternal AUC ratio, *fCmax*_*ss,LAI*_/*fCmax*_*ss,oral*_ foetal maximum plasma drug concentration ratio between oral and long-acting formulations at steady-state, *LAI* long-acting injectable, *SD* standard deviation.

^**a**^For oral doses, one dosing interval is 0–12 h.

^**b**^For LAI doses, one dosing interval is 0–4 weeks.

Predicted C:M ratios for oral and LAI aripiprazole and olanzapine are shown in Supplementary Figs. 1 and 2, respectively. Predicted mean C:M ratios at steady state within a dosing interval generally decreased with gestational age. For the oral doses, predicted C:M ratios also fluctuated within each dosing interval, rising shortly after each new dose and peaking at approximately 4 h post-dose for aripiprazole and 5 h post-dose for olanzapine, after which they plateaued until the end of the dosing interval. Similar to the oral drugs, predicted C:M ratios for both LAI drugs at steady state also decreased with gestational age, but with less fluctuations within the dosing intervals. Across trimesters and formulations, the predicted C:M ratios for both drugs were consistently below 1. Predicted median (range) C:M ratio was 0.63 (0.59-0.69) for aripiprazole and 0.77 (0.34–0.96) for olanzapine.

**Fig. 1:**
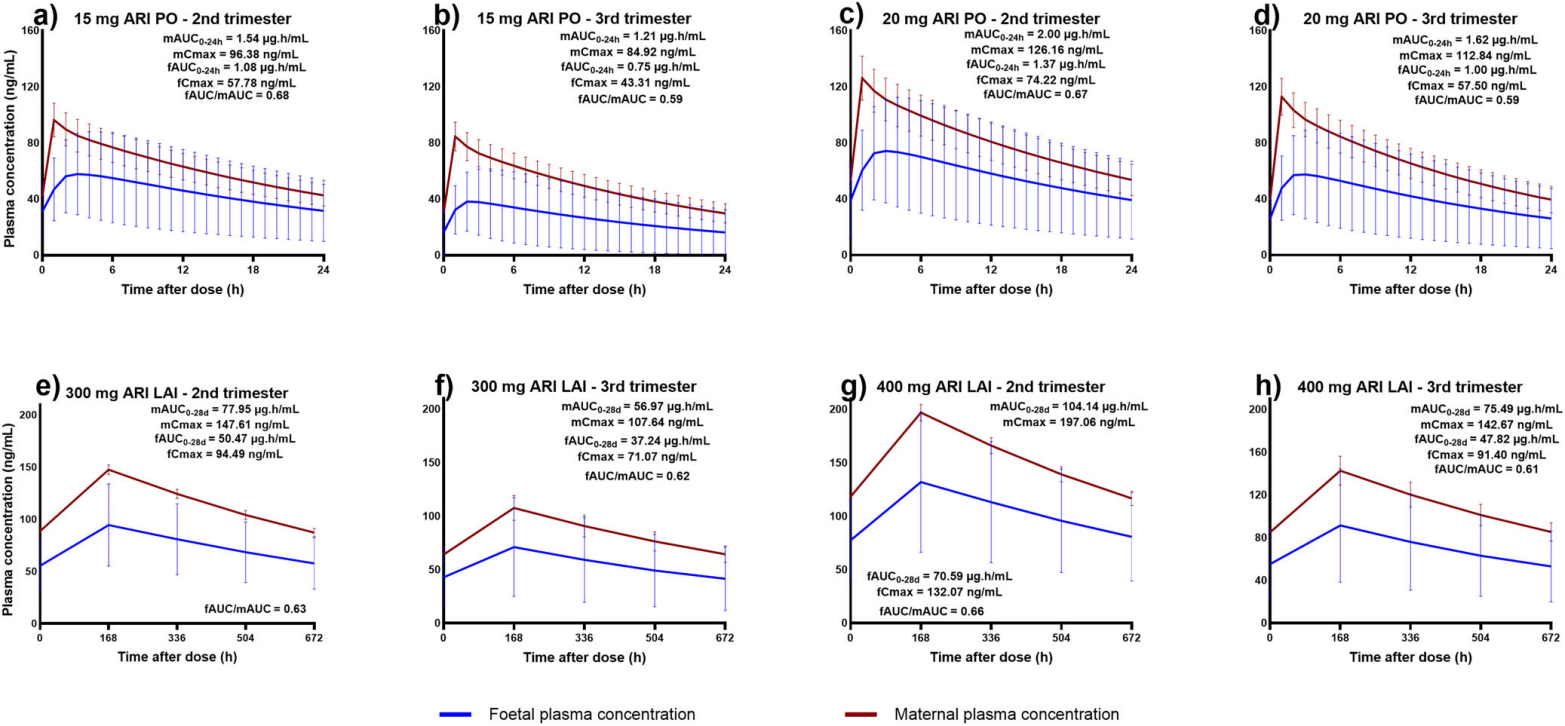
Predicted average plasma concentration-time profiles of different doses of oral aripiprazole and their therapeutic-equivalent long-acting injectable (LAI) doses in pregnancy within one dosing interval at steady-state. **a** 15 mg oral dose in the second trimester, **b** 15 mg oral dose in the third trimester, **c** 20 mg oral dose in the second trimester, **d** 20 mg oral dose in the third trimester **e** 300 mg LAI dose in the second trimester, **f** 300 mg LAI dose in the third trimester, **g** 400 mg LAI dose in the second trimester, **h** 400 mg LAI in the third trimester. ARI is aripiprazole, PO is oral, LAI is long-acting injectable, mAUC maternal area under the plasma concentration-time curve under one dosing interval, fAUC foetal area under the plasma concentration-time curve under one dosing interval, mCmax maternal maximum concentration at steady-state, fCmax foetal maximum concentration at steady state, fAUC/mAUC foetal to maternal AUC ratio.

**Fig. 2:**
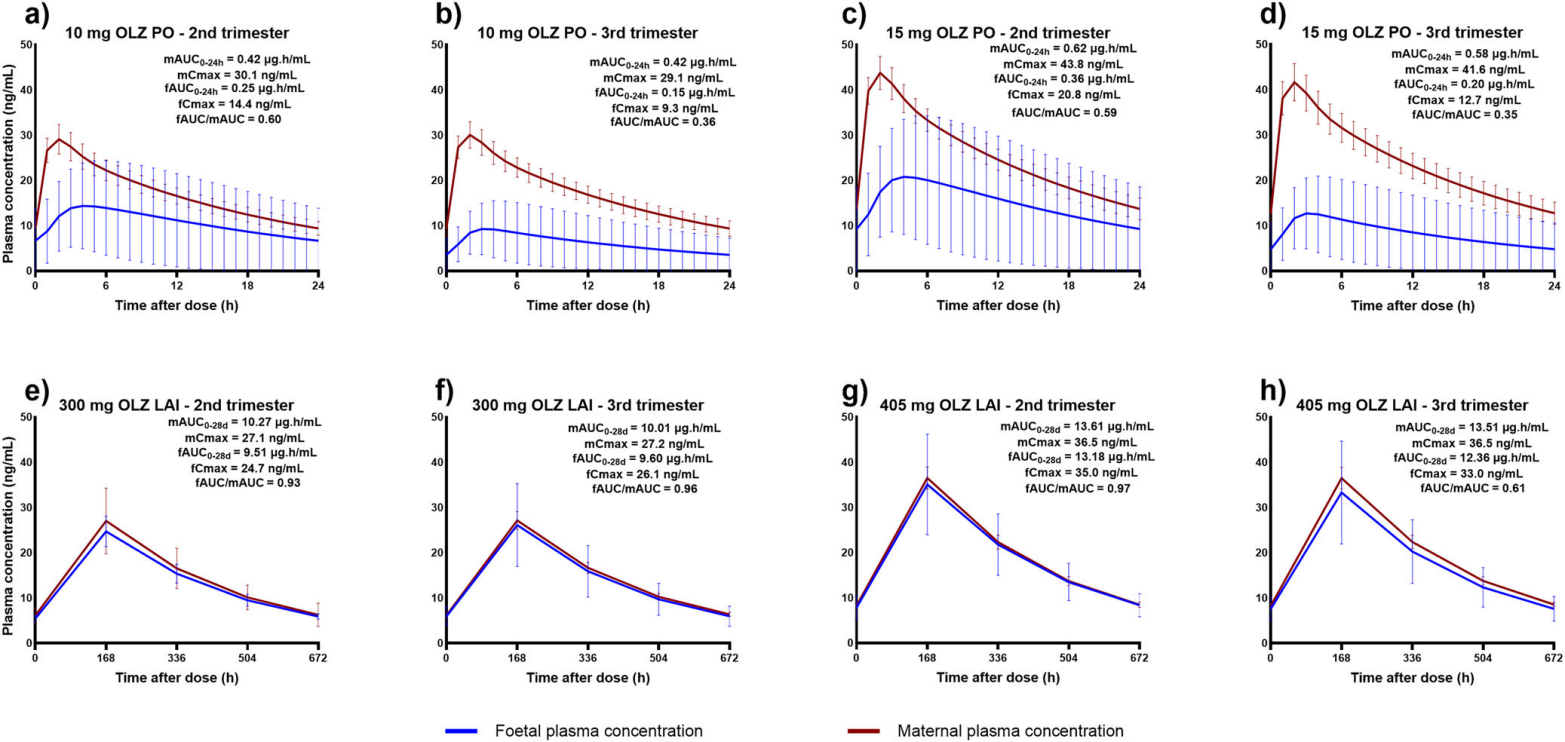
Predicted average plasma concentration-time profiles of different oral doses of olanzapine and their therapeutic-equivalent long-acting injectable (LAI) doses in pregnancy within one dosing interval at steady-state. **a** 10 mg oral dose in the second trimester, **b** 10 mg oral dose in the third trimester, **c** 15 mg oral dose in the second trimester, **d** 15 mg oral dose in the third trimester, **e** 300 mg LAI dose in the second trimester, **f** 300 mg LAI dose in the third trimester, **g** 405 mg LAI dose in the second trimester, **h** 405 mg LAI in the third trimester. OLZ olanzapine, PO is oral, LAI is long-acting injectable, mAUC maternal area under the plasma concentration-time curve under one dosing interval, fAUC foetal area under the plasma concentration-time curve under one dosing interval, mCmax maternal maximum concentration at steady-state, fCmax foetal maximum concentration at steady state, fAUC/mAUC foetal to maternal AUC ratio.

Changes in predicted maternal and foetal plasma concentrations for aripiprazole and olanzapine within a single dosing interval are shown in Figs. 1 and 2. The figures illustrate predicted foetal plasma concentrations were consistently lower than maternal plasma concentrations for both drugs, except in the case of LAI olanzapine where foetal plasma concentrations were comparable to maternal plasma concentrations in the second and third trimesters for both doses explored. In addition, the foetal exposure to both oral drugs were predicted to decrease as pregnancy progresses. For instance, the predicted mean foetal AUC for 10 mg oral olanzapine in second trimester was 0.25 μg.h/mL (7 μg.h/mL when extrapolated to 28 days) this decreased to 0.15 μg.h/mL (4.2 μg.h/mL when extrapolated to 28 days) in the third trimester, and for 20 mg oral aripiprazole it was 1.37 μg.h/mL (38.36 μg.h/mL when extrapolated to 28 days) decreasing to 1.00 μg.h/mL (28.00 μg.h/mL when extrapolated to 28 days) in the third trimester (Table 1).

## Discussion

In this study, the extent of in utero exposure was compared between the respective oral and LAI formulations. So far, only one PBPK model has been utilised to predict drug disposition of both aripiprazole^41^ and olanzapine^42^ in pregnant populations. To the best of our knowledge, this is the first study to compare in utero exposure between oral and LAI formulations of both aripiprazole and olanzapine through PBPK modelling.

Across the second and third trimesters of pregnancy, comparative analysis of oral and therapeutic-equivalent LAI formulations of aripiprazole did not show any notable difference based on the predicted C:M and fAUC/mAUC ratios (Table 1). For olanzapine, the predicted C:M and fAUC/mAUC ratios for the oral formulations were generally lower compared to their therapeutic-equivalent LAI formulations. Also, predicted C:M and fAUC/mAUC ratios for oral olanzapine were lower in the third trimester compared to the second trimester of pregnancy, unlike for LAI olanzapine which did not vary much across pregnancy (Table 1). Generally, the simulated C:M ratios consistently remained below 1 for both drugs. This suggests that irrespective of dose or formulation the portion of the drug crossing the placenta to the foetus is expected to be lower compared to the amount of the drug in the mother.

Predicted C:M ratios between oral and LAI formulations of aripiprazole, remained relatively consistent. Unlike aripiprazole, both the predicted C:M and fAUC/mAUC ratios for olanzapine varied between the two formulations. A study by Correll, et al.^43^ reported the peak-to-trough plasma concentration of oral olanzapine in comparison to its LAI once monthly formulation has an approximate 2- fold difference accounting for the disparities in PK profiles.

While C:M ratios might offer insight into the extent of foetal exposure at a specific time, it is inadequate to quantify the total amount of foetal drug exposure over time. The cumulative mean foetal AUC for the oral formulations within a 28-day period was lower for both drugs when compared to their therapeutic-equivalent LAI formulation for a similar duration (Table 1). Also, the predicted mean C_max_ decreased with gestational age except for LAI olanzapine (Table 1).

Decreasing foetal and maternal exposure to both oral and LAI aripiprazole during pregnancy could be related to gestation-dependent increase in metabolic enzyme activities and increased blood flow to the liver^44^ (Table 1). Aripiprazole is mainly metabolised by both CYP3A4 and CYP2D6. The increased activity of both metabolic enzymes would be expected to lead to increased clearance and lower exposures for aripiprazole during pregnancy^45^. Similarly, reported maternal concentrations for oral aripiprazole reduced significantly during pregnancy^45^ (Supplementary Table 2).

Though olanzapine is also metabolised by CYP3A4, unlike aripiprazole, it is mainly metabolised by CYP1A2. CYP1A2 activity decreases during pregnancy which could result in reduced clearance and higher exposures of olanzapine with increasing gestational age^44^. However, increased CYP3A4 activity during pregnancy might contribute to offsetting the expected effect of the reducing CYP1A2 activity on maternal exposure to olanzapine. This might explain the negligible difference in the clinically observed and predicted maternal exposure to olanzapine between the second and third trimesters of pregnancy^45^ (Table 1). Unlike the predicted maternal exposure to olanzapine, the predicted foetal exposure to both oral and LAI olanzapine reduced significantly across gestational age (Table 1) which could be related to increasing CYP3A4 activity that was implemented in the foetal liver. In addition, PK parameters might be affected by dosing. For instance, peak-to-plasma plasma concentrations of LAI antipsychotics may be affected by dosing^43,46^.

Zheng, et al.^42^ reported a full-body PBPK model to investigate changes in systemic exposure of oral olanzapine during pregnancy. Their predicted plasma drug concentrations did not reach the therapeutic level of 20 ng/mL required for effective treatment after the administration of 10 mg oral olanzapine. Though the predicted C_max_ achieved with oral 10 mg olanzapine in our study was well above the effective therapeutic level, overall drug plasma concentration was lower than what is needed for effective treatment in both second and third trimester. In contrast, the whole-body PBPK model utilised by Zheng, et al.^41^ predicted a gradual decline in maternal plasma drug concentrations as pregnancy progresses, a trend that was also observed in our predictions for aripiprazole. While both studies did not examine foetal exposures, our study investigated and compared extent of foetal exposure between the oral and their therapeutic-equivalent LAI formulations.

Though there has been no association between the use of oral and LAI aripiprazole with incidence of congenital malformations^47,48^, there are numerous clinical studies linking olanzapine with increased risk of musculoskeletal defects^49^. While *in utero* foetal exposure to maternal medication does not necessarily translate to risk. Direct effect on the placenta has been associated with the risk associated with some drugs. Hence, data on foetal exposure should be interpreted in a broader context that includes possible effect on the placenta and benefit-risk assessments.

The findings reported here need to be interpreted in the context of certain limitations. Contributions of placenta drug metabolism and transport which may affect the overall foetal drug exposure were not accounted for. From available literature, both aripiprazole^50^ and olanzapine^51^ are substrates of P-gp (efflux) transporters. However, there is insufficient data for proper characterisation of their roles within the model. Additionally, the current model did not capture the dehydrogenation component of aripiprazole metabolic pathway which produces dehydro-aripiprazole, a major metabolite that contributes to its pharmacological activity. Lastly, model predictions are often not exact representations of clinical data considering potential bias that could result from model overpredictions or underpredictions of PK parameters (Supplementary Tables 1-3). Thus, care should be taken when informing dose adjustments solely based on model predictions without clinical data confirming the model observations.

Model predictions for olanzapine and aripiprazole suggest that foetal exposure to these LAI antipsychotics is higher compared to their therapeutic-equivalent oral doses. Further research is needed to understand potential differences in the pharmacokinetic and safety profiles of oral formulations compared to long-acting formulations during pregnancy.

## Methods

### Model description

Our previous whole-body adult and pregnancy PBPK models developed for long-acting cabotegravir and rilpivirine, were repurposed and used for this study^52^. Both models were implemented in SimBiology®, an application within MATLAB® (version R2023b, MathWorks, Natick, USA, 2023). The pregnancy PBPK model had been extrapolated from the whole-body adult model by incorporating the various gestation-related anatomical and physiological changes, as well as pregnancy-specific compartments.

### System parameters

Anatomical and biological parameters such as organ volumes, blood flow rates, and tissue composition used in the creation of the model were obtained from available literature as earlier described^53^.

### Absorption

Oral drug absorption was modelled with the compartmental absorption and transit model^54^. The oral absorption rate constant (K_A_) was estimated using the effective drug permeability (Eq. 1), which was derived from the number of hydrogen bond donors and the polar surface area of the aripiprazole and illustrated in Eq. 2^55^. For olanzapine, K_A_ was obtained from literature^56^.

1 $${K}_{A}=\frac{2\times {P}_{{eff}}}{r}$$

2 $${P}_{{eff}}={10}^{\left(-2.546-0.011\left({PSA}\right)-0.278\left({HBD}\right)\right)}$$

where r represents the radius of small intestines.

The gut abundance^57^ and intrinsic clearance of CYP3A4^41^, were used to calculate the gut clearance (Cl_gut_) of drug. The fraction of drug escaping intestinal metabolism and reaching the liver was modelled using Eq. 3.

3 $${Fg}=\frac{{Q}_{{gut}}}{{Q}_{{gut}}+\left({{fu}}_{{gut}}\times {{Cl}}_{{gut}}\right)}$$

where fu_,gut_ is the fraction of unbound drug and is modelled as 1, and Q_gut_ (L/h) denotes the gut blood flow.

The intramuscular release of aripiprazole LAI formulation from the depot compartment in the muscle was simulated as a first-order reaction, described by Eq. 4. The release rate was obtained from literature^52^.

4 $$\frac{{d(A}_{{muscle}})}{{dt}}={-K}_{{im}}.{A}_{{im\; depot},{muscle}}$$

where d(A_muscle_)/dt, represents the aripiprazole release rate constant from the IM depot into the systemic circulation (mg/h), K_im_ denotes the IM aripiprazole rate release constant from IM depot, and A_im depot,muscle_ indicates the quantity of aripiprazole (mg) present in the IM depot within the muscle.

However, for olanzapine the intramuscular LAI release rate was better described using Eq. 5*,* which incorporated two different release rates (Supplementary Table 4). The first release rate for olanzapine was obtained from literature^58^. The other release rate was added later to improve the model performance and fitted to the model using olanzapine clinical data. However, the use of the two release rates for olanzapine was purely empirical and not informed by olanzapine literature. The model poorly predicted PK parameters for olanzapine with a single release rate.

5 $$\frac{{d(A}_{{muscle}})}{{dt}}={-K}_{{im}1}.{A}_{{im\; depot},{muscle}}-{K}_{{im}2}.{A}_{{im\; depot},{muscle}}$$

where d(A_muscle_)/dt, represents the olanzapine release rate constant from the IM depot into the systemic circulation (mg/h), A_im depot,muscle_ indicates the quantity of olanzapine (mg) present in the IM depot within the muscle.

### Distribution

**Fig. 3:**
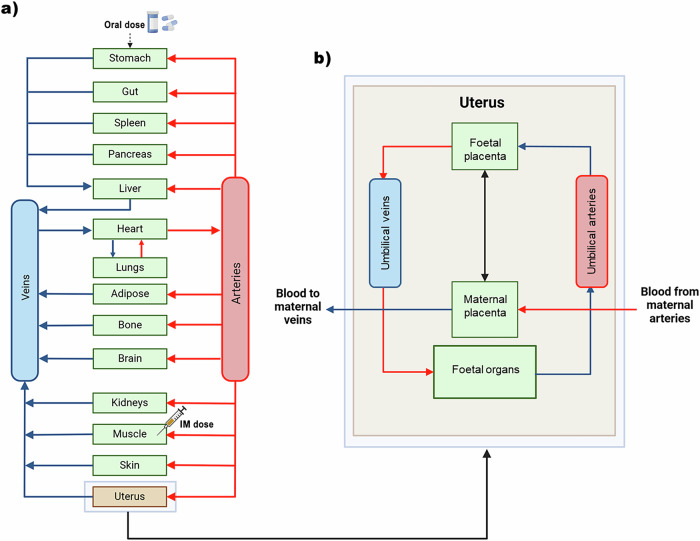
Schematic diagram of the materno-foetal physiologically-based pharmacokinetic (m-f-PBPK) model. **a** Illustration of the maternal physiologically-based pharmacokinetic (PBPK) model representing tissues and organs as compartments with the arrows indicating the direction of blow flow. **b** Schematic diagram of the foetal compartment as modelled in the uterus. (Adapted from Atoyebi, et al.^37^ and recreated on Biorender.com). IM: intramuscular.

The schematic diagram of the pregnancy PBPK model is illustrated in Fig. 3a showing the foetal component within the uterus. Key model assumptions included perfusion-limited drug distribution and the well-stirred distribution^37^. Volume of drug distribution (V_ss_) was modelled using the tissue-to-plasma ratios and volumes of the various compartments^59^. The impact of pregnancy on the fraction of unbound drug was modelled using equations from existing literature^60,61^.

### Metabolism

Aripiprazole metabolism was modelled as previously reported in literature^41^. Based on in-vitro studies, aripiprazole undergoes three biotransformation pathways mediated by CYP2D6 and CYP3A4: hydroxylation, N-dealkylation and dehydrogenation ( https://www.accessdata.fda.gov/drugsatfda_docs/label/2014/021436s038,021713s030,021729s022,021866s023lbl.pdf). Therefore, the total liver clearance was modelled using reported drug clearances by each enzyme^41^. Thus, the liver clearance was modelled using Eq. 6.

6 $${{Cl}}_{{hep},{ARI}}=\left(\frac{{{Cl}}_{\mathrm{int}}{CYP}3A4}{{f}_{m}{CYP}3A4}\right)+\left(\frac{{{Cl}}_{\mathrm{int}}{CYP}2D6}{{f}_{m}{CYP}2D6}\right)$$

where Cl_int_CYP3A4 is the intrinsic clearance by the CYP3A4 enzyme; Cl_int_CYP2D6 is the intrinsic clearance by the CYP2D6 enzyme; Cl_hep,ARI_ is the total liver clearance of aripiprazole,

f_m_CYP3A4 is the fraction metabolised by CYP3A4 and f_m_CYP2D6 is the fraction metabolised by CYP2D6.

For olanzapine metabolism, liver clearance was determined based on the intrinsic clearance of the drug by the following enzymes: CYP1A2, CYP2C8, CYP3A4, FMO3, and UGT1A4^62^. The intrinsic clearance of the drug per milligram of microsomal protein for each enzyme was scaled to the entire liver, accounting for the microsomal protein content as well as total liver weight (Eqs. 7–8). However, the renal olanzapine clearance was not included in the model.

7 $$\begin{array}{c}C{l}_{int{,}OLZ}{=}C{l}_{int}CYP{1}A{2}{+}C{l}_{int}CYP{2}C{8}{+}C{l}_{int}CYP{3}A{4}\\ {+}C{l}_{int}FMO{3}{+}C{l}_{int}UGT{1}A{4}\end{array}$$

8 $${{Cl}}_{{hep},{OLZ}}={{Cl}}_{\mathrm{int},{OLZ}}\times {MPPGL}\times {{Weight}}_{{liver}}$$

Where Cl_int,OLZ_ is the total intrinsic clearance of olanzapine; Cl_int_CYP1A2 is the intrinsic clearance by the CYP1A2 enzyme; Cl_int_CYP2C8 is the intrinsic clearance by the CYP2C8 enzyme; Cl_int_CYP3A4 is the intrinsic clearance by the CYP3A4 enzyme; Cl_int_FMO3 is the intrinsic clearance by the FMO3 enzyme; Cl_int_UGT1A4 is the intrinsic clearance by the UGT1A4 enzyme; Cl_hep,OLZ_ is the total liver clearance of olanzapine; MPPGL is the weight of microsomal protein per gram of liver and, Weight_liver_ is the total weight of the liver (kg).

Fraction of drug escaping the liver into the systematic circulation (Fh) was determined by using the total liver clearance (Cl_hep_), as described in Eq. 9.

9 $${F}_{h}=\frac{{Q}_{h}}{{Q}_{h}+\left({{Cl}}_{{hep}}\times \frac{{f}_{{up}}}{R}\right)}$$

where Q_h_ denotes hepatic blood flow rate, f_up_ represents the fraction of unbound aripiprazole and olanzapine in plasma and R indicates the tissue-to-plasma ratio of drug in the liver.

### Pregnancy PBPK model

The whole-body pregnancy PBPK model was similar to the pregnancy PBPK model reported by Atoyebi, et al.^37^. The development of the pregnancy PBPK model included involved feminising the adult PBPK model by restricting gender-specific parameters to reflect female-only characteristics. Additionally, the model was modified to incorporate pregnancy-induced anatomical and physiological changes known to influence drug disposition^44,63^. The rate of blood flow to various tissues and organs were calculated as fractions of cardiac output, using data from available literature^63^. Furthermore, gestation-related changes to the metabolic activity of CYP3A4 and CYP2D6 as reported by Abduljalil, et al.^44^, were implemented into the model for aripiprazole, Eqs. (10–11).

10 $${CYP}3A4{activity}\,\left( \% \right)=100+2.9826{GA}-0.0741{{GA}}^{2}$$

11 $${CYP}2D6{activity}\,\left( \% \right)=100+2.2695{GA}-0.0348{{GA}}^{2}$$

Where GA denotes gestational age. With regards to olanzapine pregnancy PBPK model, changes to metabolic activity of CYP1A2, CYP3A4, UGT1A4 and FMO3 were accounted by calculating fold-change throughout each trimester based on values reported by Korprasertthaworn, et al.^62^ and Zheng, et al.^42^. CYP2C8 activity was assumed to be consistent throughout pregnancy due to insufficient quantitative information for characterisation^42^.

### Drug parameters

The PBPK models were refined to simulate both aripiprazole and olanzapine metabolism by incorporating key drug-specific parameters reported in literature. These parameters included the acid dissociation constant, the octanol-water partition coefficient, and the first-order release rate constant for the intramuscular depot, which play significant roles in simulating drug absorption and distribution. Presented in Supplementary Table 4 is an overview of the parameters, including both the in vitro parameters and physico-chemical properties of aripiprazole and olanzapine implemented in the models.

### Validation of PBPK models with clinical data

For each scenario, simulations were conducted in 100 virtually healthy population with dosages, routes of administrations, and demographics, set as reported in the clinical studies used for model validation. Predicted drug concentration-time data were analysed for dosing intervals of interest for each simulation and used to determine pharmacokinetic (PK) parameters, including the area under the curve (AUC_t_), maximum concentration (C_max_), minimum concentration (C_min_), and half-life (T_1/2_). Predicted PK parameters were compared against observed data reported in the clinical studies. A prediction error within a 2-fold range was considered the acceptable limit for this study, accounting for the any variability in predicted PK parameters, as outlined by Abduljalil, et al.^64^.

### Aripiprazole

For validation of the whole adult PBPK model for aripiprazole, clinical data from non-pregnant populations receiving oral and LAI aripiprazole were used. For the oral formulation, clinical PK data from 5 mg and 10 mg single oral dose of aripiprazole were used, as reported by Boulton, et al.^65^ in 14 participants over 384 h and by Wojnicz, et al.^66^ in 103 healthy volunteers over 72 h, respectively. Similarly, clinical PK data from Raoufinia, et al.^38^ were used for the validation of LAI aripiprazole, specifically focusing on single and repeated monthly administrations of 400 mg AOM administered via both gluteal and deltoid injections in patients with schizophrenia. In the single administration study, both cohorts receiving the 400 mg aripiprazole dose via deltoid (*n* = 18) and gluteal (*n* = 19) administration were monitored for over 126 days. For the repeat-administration study, participants were observed for 141 days, with two different administration sequences: deltoid/deltoid (*n* = 73) and gluteal/deltoid (*n* = 68). All steady-state PK samplings in the repeat-dose study were conducted after the fifth injection.

Due to the limited availability of clinical data in pregnant populations, no literature specifically reported the PK of aripiprazole in either oral or LAI, in this group. Instead, timepoint concentrations for aripiprazole in pregnant women for each trimester, as reported by Westin, et al.^45^, were used to validate the pregnancy model. The participants (*n* = 14) were on 15 mg once daily aripiprazole until the end of term and had reached steady-state concentrations at the time of PK sampling.

### Olanzapine

The adult model was validated with clinical PK data for olanzapine reported by Du et al.^67^, where a single dose of 5 mg was orally administered to healthy volunteers under fed (*n* = 24) and fasting (*n* = 30) conditions. For validation of single dose of 10 mg, clinical PK data used was reported by Sun et al.^68^. which included healthy male and female volunteers (*n* = 45) observed for a period of 168 h. For LAI, clinical data used were from 300 mg bi-weekly injections (*n* = 19) and 405 mg monthly injections (*n* = 29) of olanzapine in patients with schizophrenia over a period of 24 weeks as reported by Mitchell et al.^69^

For the validation of the pregnancy PBPK model for olanzapine, clinical data used were drug plasma concentration-time data reported by Westin et al.^45^ for orally administered 10 mg olanzapine in patients with psychosis (*n* = 29) across all three trimesters during pregnancy.

### Foetal compartment modelling

A multi-compartment foetal sub-model was integrated into the uterus compartment to create the materno-foetal PBPK (m-f-PBPK) model. It includes compartments representing maternal-placenta, foetal-placenta, foetal liver, rest of the foetus, umbilical veins and umbilical arteries (Fig. 3b).

Drug movement within the foetal sub-model was implemented as following a defined path where drugs within the maternal bloodstream were delivered to the maternal side of the placenta via the maternal arteries. At the placenta, drugs cross the placental barrier and exchange with the foetal blood. This exchange via passive diffusion allows drug transfer to the foetal side of the placenta. From there, the drugs are carried through the umbilical vein towards the foetal organs, passing through the foetal ductus venosus and the foetal portal sinus, where they are distributed to the foetal tissues. After circulating through the foetal organs, the remaining drugs in the blood, is collected via the umbilical arteries and carried back to the placenta. At the placenta, another exchange occurs across the placental membrane, allowing the available drugs to transfer to the maternal side via passive diffusion. Finally, these substances transferred back to the maternal veins for excretion and or further metabolism by the maternal body.

Blood flow to foetal organs was modelled using equations described by Zhang et al.^36^. The equation defining blood flow through the portal sinus was parameterised as the difference between the blood flow in the ductus venosus and the umbilical vein. The blood flow through the foetal hepatic vein was modelled as the total blood flow through foetal hepatic vein and foetal portal vein. Similarly, the blood flow through the foetal liver was characterised by the summation of the blood flow through the foetal hepatic vein and foetal portal sinus. Presented in Table 2 are equations of the various gestation-related changes to blood flows in the various blood vessels, as well as various foetal organs implemented in the foetal model^36,44^.

**Table 2:** Equations for blood flows and organ measurements used in the foetal model

**Blood flows:**
$$\begin{array}{c}{Foetal\; portal\; vein}=0.714+0.0489{GA}+0.0008\,{{GA}}^{2}\end{array}$$
$$\begin{array}{c}{Ductus\; venosus}=2.05-0.297{GA}+0.0116\,{{GA}}^{2}\end{array}$$
$$\begin{array}{c}{Foetal\; umbilical\; vessel}=0.647-0.227{GA}+0.0179G{A}^{2}\end{array}$$
**Foetal organs**^a^**:**
$$\begin{array}{c}{Foetal\; liver}=\frac{16.6-2.92{GA}+0.143G{A}^{2}}{1000}\end{array}$$
$$\begin{array}{c}{Foetal}-{Placenta}=0.5\times \left(0.0-\frac{0.716{GA}+0.9149G{A}^{2}-0.0122G{A}^{3}}{1000}\right)\end{array}$$
$$\begin{array}{c}{Maternal}-{Placenta}=0.5\times \left(0.0-\frac{0.716{GA}+0.9149G{A}^{2}-0.0122G{A}^{3}}{1000}\right)\end{array}$$
$$\begin{array}{c}{Foetus\; compartment}=0.01\,\times \frac{\left(exp\left(\left(\frac{0.955}{0.0702}\right)\left(1-exp\left(-0.0702{GA}\right)\right)\right)\right)}{1000}\end{array}$$

^a^Other parameterised foetal organs include umbilical veins and umbilical arteries; however, they were modelled as 1. GA – gestational age (in weeks).

The bidirectional passive diffusion of the drugs (Q_pd_) across the placenta was established as described by Zhang, et al.^36^. The diffusion rate across the placenta Eq. 12 was quantified by adapting Fick’s law of diffusion^36,70^.

12 $${Q}_{{pd}}=\frac{K\times {{SA}}_{{pv}}\times {f}_{u}\times \left({C}_{1}-{C}_{2}\right)}{{PT}}$$

13 $$S{A}_{{pv}}=4.66-0.788{GA}+0.0383\,G{A}^{2}-0.0004G{A}^{3}$$

14 $${PT}=\left(0.9766{GA}+3.4146\right)\times {10}^{-3}$$

where K denotes the diffusion rate constant, which was fitted to 17 for aripiprazole and 0.0108 for olanzapine. SA_pv_ represents the surface area of the placental villous (m^2^), f_u_ is the fraction of the unbound, (C_1_ - C_2_) denotes the transplacental concentration gradient, PT represents the thickness of the placenta (m), and GA represents gestational age (weeks).

Foetal liver clearance was parameterised by including CYP enzymes present in the foetal liver using Eq. 14. The foetal intrinsic clearance of CYP3A4 was extrapolated from the adult intrinsic clearance values. Additionally, the foetal abundance of CYP3A4, microsomal mass per gram of foetal liver, and foetal liver weight were all sourced from available literature^63,71,72^.

14 $${Foetal}\,{{Cl}}_{{hep}}={{Cl}}_{\mathrm{int}}{CYP}3A4\times {Abundanc}{e}_{{fetal}}{CYP}3A4\times {MMPG}{L}_{{fetal}}$$

Where Cl_int_CYP3A4 is the adult intrinsic clearance by the CYP3A4 enzyme, Abundance_foetal_CYP3A4 is the foetal abundance of CYP3A4, and MMPGL_foetal_ is the microsomal mass per gram of foetal liver (mg/g).

### Validation of the m-f-PBPK model

Using the m-f-PBPK model, simulations were performed to validate the model predictions of drug transfer to the foetal compartment. For aripiprazole, these simulations were compared against a case study reported by Nguyen et al. (2011), involving a 27-year-old mother with schizophrenia who was administered 10 mg of the drug at 37 weeks of gestation and was considered to have reached steady-state at delivery (39.3 weeks), the time of PK sampling. To validate the maternal-foetal dyad for olanzapine, a single case study reporting foetal and maternal concentration at delivery was also used^73^. In accordance with the acceptance criteria implemented for this study, the pregnancy model satisfactorily predicted foetal exposure to oral aripiprazole and olanzapine in the pregnant population across all three trimesters and at delivery. Thus, it was assumed that model predictions for foetal exposure to LAI drugs would also be probable.

### Modelling foetal exposure to oral versus LAI formulations

Reported oral aripiprazole dosages of 15 mg and 20 mg have shown PK profiles comparable to those of LAI aripiprazole at 300 mg and 400 mg, respectively^38,39^. Additionally, the most recommended starting oral dose for olanzapine is 10–15 mg once daily, with corresponding LAI doses being 300 and 405 mg once monthly respectively^42,74,75^. Thus, simulations were conducted to predict the pharmacokinetics of the oral dosages (e.g., 15 mg ARI and 15 mg OLZ) and their therapeutic-equivalent LAI doses (300 mg ARI and 405 mg OLZ, respectively) in virtual pregnant populations (*n* = 100 per scenario). The mean (SD) body weight and age of the virtual population were 57.9 (12.6) kg and 31.5 (7.9) years, respectively.

For oral doses, one dosing interval (within 24 h) at steady state was analysed during the second and third trimesters. Similarly, steady-state pharmacokinetics of LAI aripiprazole were simulated during the second and third trimesters with analysis carried-out for a monthly dosing interval.

Foetal exposure was estimated using the m-f-PBPK model, and calculations were based on time-averaged cord plasma concentration relative to maternal plasma concentration ratio (C:M ratio). Furthermore, the foetal-to-maternal exposure ratio within each dosing interval was estimated by dividing the area under the drug concentration curve (AUC) of the foetus by that of the mother (fAUC/mAUC). Variations in foetal and maternal plasma concentrations over one dosing interval at steady state were also assessed.

## Supplementary information

Supplementary information

## Data Availability

Data is provided within the manuscript and supplementary information files. The datasets generated and/or analysed during the current study are also available from the corresponding author upon reasonable request. In addition, the related model file (code) developed for this study can be obtained from the corresponding author upon reasonable request.

## Code availability

Related model file developed for this study can be obtained from the corresponding author upon reasonable request.
